# Supplementary material for: Effect of prenatal alcohol consumption on dental enamel formation in offspring—An animal study protocol
Source: PLoS One. 2025 Feb 14;20(2):e0317570. doi: 10.1371/journal.pone.0317570 (PMC11828354; doi:10.1371/journal.pone.0317570)
Supplement: Attachment — Submitted filename: Response to Reviewers.docx [file pone.0317570.s001.docx]

July 12^th^, 2024

**Dr. Boyen Huang**, *DDS, MHA, PhD*

Academic Editor

PLOS ONE

**PONE-D-24-17362**

**Effect of prenatal alcohol consumption on dental enamel formation in offspring – an animal study protocol**

Dear Editor,

Thank you very much for your message. We are submitting a revised version of our manuscript after addressing the areas that required clarification, as mentioned by the reviewers. We appreciate the valuable suggestions to our manuscript. Additional information has been added/suppressed/modified as requested.

Please find our answers below. We hope our corrections are appropriate and that the manuscript may be reconsidered for publication. Please let us know if you have any questions.

Thank you very much for your consideration.

Warm regards,

The Corresponding author and co-workers

**Journal Requirements:**

When submitting your revision, we need you to address these additional requirements.

1. Please ensure that your manuscript meets PLOS ONE's style requirements, including those for file naming. The PLOS ONE style templates can be found at 
<https://journals.plos.org/plosone/s/file?id=wjVg/PLOSOne_formatting_sample_main_body.pdf> and 
<https://journals.plos.org/plosone/s/file?id=ba62/PLOSOne_formatting_sample_title_authors_affiliations.pdf>

**Authors:** We have ensured that the current version of the manuscript meets the journal's style requirements, in accordance with the PLOS ONE style templates indicated above.

2. We note that the grant information you provided in the ‘Funding Information’ and ‘Financial Disclosure’ sections do not match.

When you resubmit, please ensure that you provide the correct grant numbers for the awards you received for your study in the ‘Funding Information’ section.

**Authors:** The grant information in the "Funding Information" and "Financial Disclosure" sections now matches. We have made sure to provide the correct grant/scholarship numbers for the awards in the 'Funding Information' section.

3. Thank you for stating the following financial disclosure: 
"The author(s) received no specific funding for this work."

At this time, please address the following queries:

a) Please clarify the sources of funding (financial or material support) for your study. List the grants or organizations that supported your study, including funding received from your institution.

b) State what role the funders took in the study. If the funders had no role in your study, please state: “The funders had no role in study design, data collection and analysis, decision to publish, or preparation of the manuscript.”

c) If any authors received a salary from any of your funders, please state which authors and which funders.

d) If you did not receive any funding for this study, please state: “The authors received no specific funding for this work.”

Please include your amended statements within your cover letter; we will change the online submission form on your behalf.

**Authors:** The 'Financial Disclosure' section was modified. The queries above (points a-d) were addressed.

A cover letter indicating our amended statements was submitted.

4. We note that Figure 1 in your submission contain copyrighted images. All PLOS content is published under the Creative Commons Attribution License (CC BY 4.0), which means that the manuscript, images, and Supporting Information files will be freely available online, and any third party is permitted to access, download, copy, distribute, and use these materials in any way, even commercially, with proper attribution. For more information, see our copyright guidelines: <http://journals.plos.org/plosone/s/licenses-and-copyright>.
We require you to either (1) present written permission from the copyright holder to publish these figures specifically under the CC BY 4.0 license, or (2) remove the figures from your submission:

a. You may seek permission from the original copyright holder of Figure 1 to publish the content specifically under the CC BY 4.0 license.

We recommend that you contact the original copyright holder with the Content Permission Form (<http://journals.plos.org/plosone/s/file?id=7c09/content-permission-form.pdf>) and the following text:

“I request permission for the open-access journal PLOS ONE to publish XXX under the Creative Commons Attribution License (CCAL) CC BY 4.0 (<http://creativecommons.org/licenses/by/4.0/>). Please be aware that this license allows unrestricted use and distribution, even commercially, by third parties. Please reply and provide explicit written permission to publish XXX under a CC BY license and complete the attached form.”

Please upload the completed Content Permission Form or other proof of granted permissions as an ""Other"" file with your submission.

In the figure caption of the copyrighted figure, please include the following text: “Reprinted from [ref] under a CC BY license, with permission from [name of publisher], original copyright [original copyright year].”

b. If you are unable to obtain permission from the original copyright holder to publish these figures under the CC BY 4.0 license or if the copyright holder’s requirements are incompatible with the CC BY 4.0 license, please either i) remove the figure or ii) supply a replacement figure that complies with the CC BY 4.0 license. Please check copyright information on all replacement figures and update the figure caption with source information. If applicable, please specify in the figure caption text when a figure is similar but not identical to the original image and is therefore for illustrative purposes only.

**Authors:** Figure 1 was modified, excluding the copyrighted images.

**Reviewer comments**

**Reviewer #1 - Dr. Devi Atukorallaya**

The article entitled “Effect of prenatal alcohol consumption on dental enamel formation in offspring – an animal study protocol" by Leme et al. proposed to analyze the rat tooth enamel development with the embryonic alcohol exposure. This is a novel research area which tried to investigate the effect of alcohol on causing developmental defects of the enamel.
They are using various methods to investigate the structure of the enamel and tried to identify the underlying protein gene activity in causing these enamel defects.
I really enjoyed reading the proposed project and I have following comments to improve the content of this protocol.

1. Under the animals and experimental procedures
Exposure to alcohol will be carried out following a binge drinking model, in which the animals will receive doses of ethanol via gavage (3g/Kg, [30% w/v]) for 3 consecutive days followed by 4 days of rest per week [33, 34]. The animals of the control group will receive distilled water. After one week of ethanol exposure ….
Explain the rationale of using this alcohol concentration. The cited two references have similar concentrations what is the rationale for using the (30% W/V) versus 1- 5% of alcohol concentrations.

**Authors:** Thank you very much for the positive evaluation of our manuscript. Below are the responses to your comments.

We are providing the rationale for the binge drinking model used (please see the 2nd and 3rd paragraphs of the 'Animals and experimental procedures' section). We decided to use the indicated alcohol dose based on previous literature, with the objective of achieving the blood alcohol concentration that characterizes a heavy/binge drinking pattern.

2. The figure 2 has a confusion on left hemi mandible – Are you going to use the incisors and molars both for Rt-PCR and micro-CT? How you are going to do that? Please explain.

**Authors:** Thanks for your comment. We plan to perform Rt-PCR and micro-CT, both on the lower incisors. For evaluation with micro-CT, the part of the tooth just above the bone ridge will be sectioned, to perform measurements previously made by our team (Ref. 44 of the manuscript). The intraosseous section of the tooth will be used to collect ameloblasts following a previously published protocol (Ref. 46 of the manuscript). The molars, as indicated in Fig 2, will only be used for micro tomographic evaluations.

We added information to the 'Micro-computed tomography' and 'Real-time reverse transcription polymerase chain reaction' sections for better understanding of the readers.

3. In the histological evaluations under histometric analysis “Microscope will be operated in fluorescence mode” Why you need the fluorescence mode as these sections will be H &E sections.

**Authors:** Thank you for your comment. We apologize for the mistake. In fact, in this case it is not necessary to use the microscope in fluorescence mode. Bright-field microscopy will be used for these analyses. This information was corrected in the 'Histometric analysis' section.

4. Please indicate the data availability according to the journal policy.

**Authors:** Thank you very much for your comment. We review the journal's data availability policy. Follow what is indicated:

***What if my article does not contain any data?***

*All articles must include a Data Availability Statement but some submissions, such as Registered Report Protocols and Lab or Study Protocol articles, may not contain data. For manuscripts that do not report data, authors must state in their Data Availability Statement that their article does not report data and the data availability policy is not applicable to their article.*

(<https://journals.plos.org/plosone/s/data-availability>)

Therefore, we stated the following: ''This article does not report data and the data availability policy is not applicable".

**Reviewer #2 – Dr. Sylvie Babajko**

Roberta Duarte Leme and co-workers propose a binge drinking experimental model to study the effects of 3g/Kg alcohol [30% w/v] in pregnant rats on dental development of the pups.

The introduction part should be re-organized:

1- rationale of the study

2- State-of-the-art of alcohol effects on dental development.

The paragraph on general effects of alcohol on embryogenesis should be reduced as it is out of the scope.

References should be up-dated by avoiding papers written in non-English language.

Authors should distinguish studies on humans and on animals.

**Authors:** Thank you very much for your valuable appreciations. Below are our responses to your comments.

We re-organized the introduction as suggested. Although we reduced the paragraph on general effects of alcohol on embryogenesis, we decided to maintain some information as we consider it provides an adequate background/rationale for the present study. The references were updated, using suggestions from articles recommended by you, and we avoided the use of non-English language manuscripts. We appropriately indicate in which sample each referenced study was carried out (in humans or in experimental animal models).

Figure 1: authors should add the time of exposure to alcohol.

**Authors:** Thank you for your comment. The time of exposure to alcohol was added in Fig 1.

Animals: Doses, time and duration of exposure should be justified.

**Authors:** Thank you for your comment. The rationale for the alcohol exposure protocol used was provided (please see the 2nd and 3rd paragraphs of the 'Animals and experimental procedures' section).

Overall, investigations of 10-days-old rats (PND 10) should be revised to the fact that. Neither incisors nor molars are fully erupted.

**Authors:** Thanks for your comment. We agree that both teeth have not fully erupted in 10-day-old rats. In fact, the molars are still developing intrabony during this phase.

The incisors emerge through the gum between 8 and 10 days after birth (Addison and Appleton, 1915; Schour and Massler, 1949), while the molars do so around day 19 (Schour and Massler). At 28 days, approximately one week after weaning, the first and second molars are already in occlusion for a few days (Denes et al., 2018).

Our objective in choosing the evaluation moments indicated in the protocol is to evaluate the enamel in its different stages: pre-eruptive (molars at 10 days), eruptive (incisor at 10 days) and post-eruptive and in function (incisors and molars at 28 days).

Specifications were added in the fourth paragraph of the 'Animals and experimental procedures' section.

Photography: not sure that authors could see some defects on incisal enamel due to the very short time of adversity and incomplete development at PND 10.

**Authors:** Thank you for your comment. We agree with your comment. However, we chose to maintain these evaluations in the protocol for clinical recording of the enamel of recently erupted incisors (see response to the previous comment).

Knoop microhardness test: it would be more appropriate to investigate enamel hardness by nano-indentation rather micro-indentation due to the small size of samples.

**Authors:** Thanks for your comment. Although we understand your suggestion, for logistical reasons, we have chosen to maintain enamel microhardness evaluations using the Knoop microhardness test. Despite the small size of the samples, our team has already successfully carried out the evaluation of the microhardness of the enamel of rodent incisors with this method (Ref. 44 of the manuscript).

Histology: Authors should justify why “bone blocks containing incisives and molars will be removed from the previously dissected mandibles and maxillae.”

**Authors:** Thanks for your observation. We made some modifications in this regard in the manuscript. The phrase mentioned (section 'Histometric analysis') makes sense for histological evaluations (i.e., histometric analysis, immunohistochemistry and *in situ* zymography). In this case, the incisors and molars will not be separated from their surrounding bone to avoid damaging the enamel that will be evaluated.

In the case of Rt-PCR evaluations, which will be performed only on lower incisors, we will follow a previously reported protocol for collecting ameloblasts (Ref. 46 of the manuscript); therefore, the teeth will not be removed with their surrounding bone. This last specification was added in the 'Real time reverse transcription-polymerase chain reaction' section.

Real time reverse transcription-polymerase chain reaction (RT-PCR): not sure that authors will have enough material for RT-PCR analysis especially at PND 10.

**Authors:** Thank you for your important observation. We will follow a previously published protocol (Ref. 46 of the manuscript) for appropriate collection of ameloblasts and obtaining enough total RNA for the analyses. We already have performed pilot studies in our laboratory and could collect enough amount of total RNA to evaluate gene expression.

This information was added in the 'Real time reverse transcription-polymerase chain reaction' section. Considering that at PND 10 the lower incisors will have already emerged into the oral cavity, we assume that ameloblast collection, following the indicated protocol, will be possible.

Target genes and proteins: authors should add MMP2 and MMP9 to be able to compare their data with zymography

**Authors:** Thank you for your important observation. MMP2 and MMP9 were added as target proteins and genes for immunohistochemistry and Rt-PCR analyses, respectively (please see sections 'Immunohistochemistry' and 'Real time Reverse Transcription-polymerase chain reaction').

Figure 2 should be corrected in accordance with modifications of the protocol.

**Authors:** Despite the modifications made according to the suggestions, we consider that the protocol for outcome evaluations will be maintained. Therefore, Fig 2 was not modified.

Is it possible to add a paragraph on expected data?

**Authors:** We added a paragraph on expected results (please see 'Expected results' section after the 'Statistical analysis' section).

Abstract should be revised according to modifications of the paper.

**Authors:** The abstract was revised according to the modifications made in the paper.

Reference list should be completely revised. Main studies reporting alcohol effects on dental development are quite old, authors should up-date the reference list and include recent papers (de Carvalho P, et al., 2022; Blanck-Lubarsch M et al., 2019; etc…). They should add the important review of Silva MJ et al., 2016 reporting an association between alcohol consumption and MIH.

**Authors:** The reference list was revised. Recent papers on the alcohol effects on dental development were added as suggested.
