# Supplementary material for: Effect of prenatal alcohol consumption on dental enamel formation in offspring—An animal study protocol
Source: PLoS One. 2025 Feb 14;20(2):e0317570. doi: 10.1371/journal.pone.0317570 (PMC11828354; doi:10.1371/journal.pone.0317570)
Supplement: Attachment — Submitted filename: Response to Reviewers - Plos One (Study Protocol).docx [file pone.0317570.s002.docx]

October 25^th^, 2024

**Dr. Boyen Huang**, *DDS, MHA, PhD*

Academic Editor

PLOS ONE

**PONE-D-24-17362**

**Effect of prenatal alcohol consumption on dental enamel formation in offspring – an animal study protocol**

Dear Editor,

Thank you for your message. We are submitting a revised version of our manuscript after addressing the clarifications noted by the reviewers. We appreciate their valuable suggestions. Additional information has been added, removed, or modified as requested. Please see our responses below. We hope our revisions meet your expectations and that the manuscript can be reconsidered for publication. Feel free to reach out with any questions.

Thank you very much for your consideration.

Best regards,

The Corresponding author and co-workers

**Reviewer comments**

**Reviewer #3:**

**Overall comments on the methodology**
The detail level is inconsistent method by method.

Too many sites refer to previous reports about the methodology. The authors need to present all the steps of the procedure.

All the experiments need to contain fundamental information, i.e., what types of specimens were used and how they were prepared, the number of samples, etc.

**Authors:** Thank you for your comment. The methodology section has been revised to detail and reinforce the conditions for reproducibility. All updates are highlighted in red.

There is no mention of the sample size in each experiment.

**Authors:** Thank you for your comment. The sample size for each experiment has been included in Figure 2.

The resolution of the two figures is too low to read.

**Authors:** Thank you for your comment. The images were already adjusted and verified to comply with the submission requirements, at a resolution of 600 dpi.

**Animal and experimental procedure:**
There is no mention of controlling and monitoring water and chow consumption.

**Authors:** Thank you for your comment. The animals will be subjected to a standard laboratory diet with free access to water and feed. We have added this information to the methodology section to make it clearer and more detailed.

The rationale for the sample size is not clear. How it's determined?

**Authors:** Thank you for your comment. We decided to work with an estimate of 10 offspring of each sex per group. Taking potential losses into account, this amounts to a total of 22 offspring per group. Due to the absence of prior studies on this topic, we do not have data to calculate effect sizes for each assessed outcome, making it impossible to conduct a priori sample size estimation. In this proposal, we have opted to work with 88 animals and will calculate the power of the analyses a posteriori.

The sex of the offspring should be accounted for.

**Authors:** Thank you very much for your comment. We plan to conduct the counting of males and females so that the sample is more representative, with the appropriate ratio for assessing possible sexual dimorphisms. This information has been added to the text for better understanding and is highlighted in red.

**Micro-computed tomography**
The meaning of the sentence “The region of the molars and surrounding bone will be removed for analysis” is unclear. Does it mean bone and molars will be dissected out from the mandible? If so, why is it necessary? Analyzing software can extract the preferred target region on the Micro-CT image.

**Authors:** Thank you for your comment. The incisor must be dissected to collect ameloblasts beforehand. Additionally, we decided against performing Micro-CT prior to separating the pieces to ensure that this procedure does not affect the viability of the cells.

**Scanning electron microscopy-energy dispersive X-ray**
What kind of instrument will be used to cut the tooth? How can artifacts caused by the cutting process be avoided?

**Authors:** Thank you for your valuable consideration. The incisor hemisection strategy will not be implemented. We will analyze the surface of enamel.

Any plan to etch the surface?

**Authors:** Yes, we will use phosphoric acid to etch the surface. The samples will be dried and treated with 37% phosphoric acid for 30 seconds, followed by washing with distilled water and a second drying.

**Histometric analysis**
The meaning of the sentence, “Bone blocks containing incisives (incisors?) and molars will be removed from the previously dissected mandibles and maxillae.”, is unclear.

**Authors:** Thank you for your comment. In fact, only the right hemimandibles will be used for subsequent histological processing. We have replaced any potentially confusing terms, and all updates are highlighted in red.

There is no description of what kind of structure will be observed after H&E staining.

**Authors:** Thanks for your comment. The H&E staining will be used to observe ameloblasts.

Magnification x10 would be too small to observe cells and matrix structures.

**Authors:** Thank you for your suggestion. Reviewing studies that evaluated ameloblasts in Wistar rats, we found that it may be necessary to use higher magnification levels, such as 20 and 40x. This information has been added to the text to enhance clarity.

**In situ zymography**
The procedure how to obtain the section was missing.

**Authors:** Thank you for your comment. The right hemimandibles will be dissected for subsequent analysis. The tissues will be fixed in 10% buffered formalin for 24 hours, demineralized in 5% ethylenediaminetetraacetic acid (EDTA, Merck/Millipore, Burlington, MA, USA), and then processed using standard histological techniques. To improve clarity in the text, we have separated the histological and histometric analyses, as routine histological processing is a foundational step for some of the subsequent tests.

**Real time reverse transcription-polymerase chain reaction**
The collection of the ameloblasts is not described well enough. The methods in reference #46 are not for the pure ameloblasts collection but for enamel organs enriched with ameloblasts. Authors need to be aware of it and note it.

**Authors:** Thank you for your comment. First, the incisors will be carefully isolated from the mid-mandible. Next, a brief cleaning will be performed using phosphate-buffered saline (PBS 1X), followed by scraping the surface corresponding to the ameloblasts in either the secretion or maturation phase, using an excavator or a similar tool. Finally, the cervical loop of the initiator will be removed from the apical portion. The sample can then be placed in 10% formalin or a lysis solution for further investigation. These details were included in the methodology section to improve clarity and understanding. We are aware that the methods in reference #46 are not for the collection of pure ameloblasts, and this information has been included in the methodology section to enhance clarity and reproducibility.

**Expected results**
Compared to the amount of the experiment information, expected results are too limited. Some reports use a similar method to this protocol (reference #29, 30, and DOI: 10.2485/jhtb.16.61, etc.). Therefore, the authors should be able to present the expected results of each experiment based on the previous reports.

**Authors:** Thank you for your comment. Columnar ameloblasts and odontoblasts are expected to be present, along with a reduction in the thickness of both enamel and dentin (Imai et al., 2007). Additionally, it is likely that the size of the tooth germ will be diminished. In macroscopic analyses, lesions in the enamel, identified as areas of hypomineralization or opacities, are anticipated. As a result, a decrease in mineral content is expected, which will directly affect the microhardness of the tissue. Finally, a decreased expression of key peptides involved in enamel formation and maturation is expected.

**Reviewer #4:**

This paper highlights an important area of research addressing the effects of maternal alcohol consumption on the development of enamel. Thank you to the authors for putting this paper together and working towards helping us better understand the impact of prenatal alcohol consumption on developing dentition. Some overall concerns raised by this paper are listed below:

There are multiple tests completed in this study, with many outcome evaluations completed—photography, micro-computed tomography, Knoop microhardness test, scanning electron microscopy-energy dispersive x-ray, Raman spectroscopy, histometric analysis, immunohistochemistry, in situ zymography, real time reverse transcription-polymerase chain reaction, and statistical analysis also. It is inferred that the developmental defects of enamel (DDE) will be found in offspring from rats exposed to alcohol consumption compared to those without. With 10 different DDE analysis techniques employed, no hypothesis was provided for any of the 10 tests. Without clarity on the reasoning and anticipated findings of these tests, the study lacks focus and does not have clearly defined outcomes. It seems that the tests have been assembled in an exploratory manner without a specific, well-defined objective. A ‘Study Protocol’ should have clearly defined outcomes for the physical properties (volume, thickness, and density) of enamel and cellular changes associated with alcohol consumption.

**Authors:** Thank you for your comment. The presence of columnar ameloblasts and odontoblasts is expected, along with a decrease in the thickness of both enamel and dentin (Imai et al., 2007). Moreover, it is probable that the size of the tooth germ will be reduced. Macroscopic examinations are likely to reveal lesions in enamel structure, known as areas of hypomineralization or opacities. As a result, a reduction in mineral content is anticipated, which will directly influence the microhardness and density of the tissue. Finally, a decrease in the expression of key peptides involved in enamel formation and maturation is also expected.

Another concern is that the proposal does not have any content on standardizing the tests or assessing the error. Each test described in the paper needs more detailed elaboration.

**Authors:** The evaluation of photographs will be conducted by two assessors. The Kappa correlation coefficient will be calculated to assess agreement between them. The outcomes assessed in Micro-CT and histometric analyses will be evaluated on two occasions, with a two-week interval to determine method error. Intra-evaluator repeatability will be measured using the Intraclass Correlation Coefficient, while the presence of random and systematic errors will be assessed using the Bland-Altman method. In Knoop microhardness analyses, Raman spectroscopy, and scanning electron microscopy with energy dispersive X-ray, all samples will be discarded after the tests are completed. Finally, in histological analyses, routine procedures will be conducted with precision, ensuring that the fixative is prepared in sodium phosphate-buffered solutions and that the decalcification process is properly executed, prioritizing the use of chelating agents over acids. For adequate dehydration, the volume of alcohol used should be twenty times that of the sample. During the clarification phase, the duration in xylene will be carefully monitored to prevent the material from drying out. Impregnation will take place in an oven set to 60 degrees Celsius, and during the inclusion process, the fragments will be immersed in heated paraffin to avoid the formation of air bubbles.

Power calculation should be an essential part of ‘Study Protocol’.
**Authors:** Thank you for your comment. We have chosen to estimate 10 offspring of each sex per group. Considering potential losses, this results in a total of 22 offspring per group. Due to the lack of prior studies on this topic, we do not have data to calculate effect sizes for each outcome assessed, which prevents us from performing a priori sample size estimation. In this proposal, we plan to work with 88 animals and will calculate the power of the analyses a posteriori.

Statistical testing: The description on statistical testing is inadequate. Each quantitative method should contain adequate description for statistical testing. The authors should also discuss what they will do if the underlying assumptions of ANOVA or similar tests are violated.

**Authors:** Data will be analyzed using GraphPad Prism 8.0 (Prism, Chicago, IL, USA), with a significance level set at 5%. The relative and absolute frequencies of clinical enamel alterations observed in photographic analyses, as well as enamel mineral content assessed by SEM-EDS in both groups, will be compared using the chi-square test. Continuous data from microhardness and Raman spectroscopy analyses will be compared using one-way ANOVA to evaluate the effect of alcohol. Data from microtomographic analyses (including enamel volume, thickness, and density), histomorphometric analysis (enamel matrix area), immunohistochemistry, in situ zymography, and PCR (relative mRNA expression) will be assessed using two-way ANOVA to examine the effects of alcohol, age, and their interaction. Separate analyses will be conducted for incisors and molars. If the assumptions of the tests are not met, generalized models will be employed based on the characteristics of the data.

**Additional comments:
Animals and experimental procedures**
How long is the mating period? Are female rats exposed to alcohol throughout this period?

**Authors:** Thank you for your comment. The mating period can last approximately 5 days (Rosen et al., 1987). And yes, all females will be exposed to alcohol throughout this period, as the exposure begins in the week leading up to mating and, consequently, to gestation. This information has been emphasized in the text to enhance clarity.

How long will the rats be exposed to alcohol in total? What is done if the female rat does not become pregnant in the expected mating period?

**Authors:** Thanks for your comment. In total, the females will be exposed to alcohol over a period of 4 weeks. The exposure begins in the week prior to mating. After gestation is confirmed, alcohol administration will continue for 3 weeks, following the previously mentioned Binge Drinking protocol, which consists of 3 days of intervention followed by 4 days of rest (Frazão et al., 2020). If pregnancy does not occur within the expected time frame, the rat will be removed from the experiment to prevent potential alterations in the outcome due to prior alcohol exposure.

“Binge drinking is characterized by bringing BAC up to 0.08 grams % or above, having the potential to generate signs of intoxication” – what are the signs of intoxication?

**Authors:** Thank you for your comment. Acute levels of ethanol can impair performance on tasks related to timing, response inhibition, and position discrimination (Popke et al., 2000). Additionally, symptoms such as ataxia, drowsiness, decreased consciousness, loss of reflexes, and even unconsciousness may be observed.

Please clarify for a general reader why was a binge drinking model chosen and not a lower alcohol consumption percentage.

**Authors:** We selected the Binge Drinking Model because it closely resembles current alcohol consumption patterns (i.e., it reflects reality more accurately). This type of consumption is characterized by occasional and intense episodes, differing from the chronic alcoholism model, which is less common today.

3g/kg (i.e., 3gm/1,000gm x 100%) yields a value of 0.3%, and it is difficult to determine how this equates to 30% w/v.

**Authors:** To achieve a concentration of 30% ethanol, a dilution will be prepared using 30 ml of absolute alcohol mixed with 70 ml of distilled water. This mixture will yield the desired concentration. For dosing, the target is 3 g of ethanol per kg of body weight, which equates to 3.8 ml of ethanol per kg. Assuming each animal weighs approximately 200 g, the calculated dose will be 0.6 g of ethanol, corresponding to 0.76 ml of the prepared solution.

The specific issues with some of the methods are provided as examples below:
**Photography**
Some of the key data are missing, e.g., camera angulation, room lighting, magnification, and quantitative vs qualitative analysis.

**Authors:** Based on the literature (Schmalfuss et al., 2022) and pilot projects conducted by our research group, the animals will be positioned perpendicular to the camera lens at a 90-degree angle. For ambient lighting, we will use white light for photography. Additionally, a polarized light filter will be employed. This information has been incorporated into the text for clarity.

**Micro-computed tomography**
How will the authors ensure the density measurement is accurate and standardized? Will the molars and surrounding bone be removed from the Micro-CT images or from the specimens before imaging?

**Authors:** Thank you for your comment. To ensure the accuracy and standardization of the test, the same voltage, exposure time, and data analysis parameters will be applied to all samples: 70 kV, 200 μA, Al/Cu filter of 0. 1 mm, voxel size 5.4 μm, full circle rotation steps at 0.4° angle intervals, and average scanning time about 2 h. The specimen will undergo a cutting process to isolate the incisor for the collection of ameloblasts. This strategy is intended to prevent the Micro-CT test from compromising cell viability.

“… the collected incisors and molars will be mesiodistally hemisected and one of those sections will be prepared for SEM” – was there uniformity with the selected hemi section? How will this be selected and how will the orientation be standardized?

**Authors:** Thank you for your comment. The hemisection strategy will not be implemented. After further evaluation, we determined that it would be more appropriate to reduce the number of samples for each test. This approach will help minimize potential artifacts and facilitate a more accurate analysis.

**Knoop microhardness test**
Will this test be carried out on flat or curved surfaces? What will be the orientation of the specimens/sections?

**Authors:** Thank you for your comment. Considering the natural curvature of rodent teeth, we planned to perform the test from a centralized point, aiming to achieve flat surfaces as much as possible.

Figure 2: For 10d offspring, microhardness testing will only occur for incisors and not molars. Although it is noted in the text that the molars are still under development, the figure should stand independent to the text, and this is not made clear for the general reader. There are similar issues with other tests.

**Authors:** Thank you for your comment. We revised the figure to align with the information presented in the text.

**Scanning electron microscopy-energy dispersive x-ray:**
What many spots will be examined from each specimen? Will there be outputs for other elements?

**Authors:** Typically, 3 to 5 points are examined in this test. Given the small size of the sample (rodent tooth), we plan to evaluate a minimum of 3 points. All available elements will be assessed.
