# Supplementary material for: Effect of prenatal alcohol consumption on dental enamel formation in offspring—An animal study protocol
Source: PLoS One. 2025 Feb 14;20(2):e0317570. doi: 10.1371/journal.pone.0317570 (PMC11828354; doi:10.1371/journal.pone.0317570)
Supplement: Attachment — Submitted filename: Response to Reviewers - Study Protocol.docx [file pone.0317570.s003.docx]

December 7^th^, 2024

**Dr. Boyen Huang**, *DDS, MHA, PhD*

Academic Editor

PLOS ONE

**PONE-D-24-17362**

**Effect of prenatal alcohol consumption on dental enamel formation in offspring – an animal study protocol**

Dear Editor,

We have carefully addressed all the points raised and are submitting a revised version of our manuscript. In response to the reviewers' suggestions, we have made the necessary revisions, including adding, removing, and modifying content as requested.

Please find our detailed responses to the reviewers’ comments below. We hope the revisions meet the expectations of both you and the reviewers, and that the manuscript can now be reconsidered for publication. Should you require any further clarification or have additional questions, please do not hesitate to contact us.

Thank you for your time and consideration.

Sincerely,

The Corresponding author and co-workers

**Reviewer comments**

**Reviewer #3:**

The manuscript should describe the methods in sufficient detail to prevent undisclosed flexibility in the experimental procedure or analysis pipeline, including sufficient outcome-neutral conditions (e.g. necessary controls, absence of floor or ceiling effects) to test the proposed hypotheses and a statistical power analysis where applicable. As there may be aspects of the methodology and analysis which can only be refined once the work is undertaken, authors should outline potential assumptions and explicitly describe what aspects of the proposed analyses, if any, are exploratory.

Authors: Thank you for your comment. For the SEM-EDS, RT-RAMAN, and in situ zymography outcomes, due to the lack of previous studies, these are considered exploratory analyses, and it is not possible to precisely define the expected results. Furthermore, the methods used for each procedure, such as the collection of ameloblasts, may be modified or improved as the research progresses. This information has been added to the text and is highlighted in red.

Resolution of Figures: Although the new figures are saved at 600 dpi, the letters in each figure are still too blurry and unreadable. All contents and text in the Figures need to be readable.

Authors: Thank you for your comment. The figures have been revised and adjusted on the PACE platform according to the specifications of PLOS ONE.

Collection of ameloblasts for real-time RT-PCR: As the author is aware now, the collected sample will not be homogenous with ameloblasts but enamel organs rich in ameloblasts. Therefore, it is not appropriate to describe the "collection of ameloblasts" here, even with the remark "not specifically designed for the collection of pure ameloblasts." This remark reduces the reliability of the sample collection. Instead of adding the remark, it is recommended to state that the collected sample will be enamel organs rich in ameloblasts. This way, it is more accurate in describing the sample collection.

Authors: Thank you for your comment. The information in the text has been reviewed, and the requested correction has been made.

The model of SEM is missing. This information is necessary to be consistent with other sections including information on all instruments' makes and models.

Authors: Thank you for your comment. The model has been added (JEOL JSM-6610LV) and is highlighted in red.

**Reviewer #4:**

Overall, the paper has greatly improved in quality since the previous review, thank you to the authors for their hard work. There are still areas in which can be improved, mostly around grammar. The paper requires a few more read-throughs by the authors and minor edits for comprehension. For example, “having the potential to generate signs of intoxication, impairing performance on tasks related to timing, response inhibition, and position discrimination” on page 6, and “… we will take photographs of right hemimandibles from both groups (n=22), along with incisors and molars (n=22) from the left hemimandible in each group” on page 10. The wording of both these examples can be further improved.

Authors: Thank you for your comment. The entire grammar of the project has been revised, and the necessary adjustments have been made. We appreciate the suggestion.

Please also review the paper for finer details – for example on page 6, “It differs from the chronic alcoholism model, which is less common today” – no definition of the chronic alcoholism model is provided, this comparison is consequently unclear. Additionally on page 14, “… secondary antibody for 1 hour, washed in PBS” – The acronym for PBS has not been introduced. Please review the full article to further improve readability.

Authors: Thank you for your comment. The entire article has been reviewed to further enhance readability.
